# Image quality improvement in head and neck angiography based on dual-energy CT and deep learning

**DOI:** 10.1186/s12880-025-01659-4

**Published:** 2025-04-10

**Authors:** He Zhang, Lulu Zhang, Juan Long, He Zhang, Xiaonan Sun, Shuai Zhang, Aiyun Sun, Shenman Qiu, Yankai Meng, Tao Ding, Chunfeng Hu, Kai Xu

**Affiliations:** 1 Department of Radiology, The Affiliated Hospital of Xuzhou Medical University, Xuzhou, Jiangsu Province P.R. China; 2 School of Medical Imaging, Xuzhou Medical University, Xuzhou, Jiangsu Province P.R. China; 3 Jiangsu Medical Imaging and Digital Medicine Engineering Research Center, Xuzhou, Jiangsu Province P.R. China; 4 Department of Medical Imaging, Xuzhou Medical University Affiliated Jiawang District People’s Hospital, Xuzhou, Jiangsu Province P.R. China; 5 CT Imaging Research Center, GE HealthCare China, Shanghai, China

**Keywords:** Deep learning reconstruction, Dual-energy computed tomography, Imaging reconstruction, Head and neck tomography angiography

## Abstract

**Objective:**

Compare the image quality of image reconstructed using deep learning-based image reconstruction (DLIR) and iterative reconstruction algorithms for head and neck dual-energy CT angiography (DECTA).

**Methods:**

This prospective study comprised fifty-eight patients with head and neck DECTA. Images reconstructed by four algorithms (120-kVp-like with ASIR-V40%, 50 keV with ASIR-V40%, 50 keV with DLIR-M, 50 keV with DLIR-H) were compared. CT attenuation, image noise, signal-to-noise ratio (SNR) and contrast-to-noise ratio (CNR) were all calculated. Edge rise distance (ERD) and edge-rise slope (ERS) were measured on the right common carotid artery to reflect spatial resolution. Quantitative data are summarized as the mean ± SD. The subjective image quality scores using a 5-point Likert scale were obtained for the following: overall image quality, edge sharpness of vessels, image noise, and artifacts.

**Results:**

The CT attenuation of all vessels in the 120kVp-like images were lower than the 3 sets of 50 keV images with significant difference (all *P* < 0.05). In the 50 keV images, both sternocleidomastoid muscle (SCM) and white matter (WM) had a minimum noise in DLIR-H group, and a maximum in ASIR-V40% group with significant difference (all *P* < 0.001). SNR and CNR in 50 keV images of all vessels had the same results: highest in DLIR-H group and lowest in ASIR-V40% group with significant differences (all *P* < 0.05). The mean value of ERD showed no significant difference among the four groups (*P* = 0.082). While the 120kVp-like images had the lowest ERS, which showed statistically significant difference with the other groups (all *P* < 0.001). In terms of overall image quality, sharpness, and artifacts, the scores of DLIR-M and DLIR-H at 50 keV were not statistically different (all *P* > 0.05), and were higher than ASIR-V40% at 50 keV images (all *P* < 0.05), and higher than ASIR-V40% at 120 kVp-like (all *P* < 0.05). The scores of DLIR-H at 50 keV were highest in terms of noise and average scores.

**Conclusion:**

DLIR is a potential solution for DECTA reconstruction since it can greatly reduce image noise, improving image quality of head and neck DECTA at 50 keV It is worth considering adopting in routine head and neck CTA applications.

**Supplementary Information:**

The online version contains supplementary material available at 10.1186/s12880-025-01659-4.

## Introduction

Head and neck computed tomography angiography (CTA) has long been considered as a noninvasive and robust imaging technique characterized by high spatial resolution. It facilitates rapid assessment of intracranial and cervical vessels, and is crucial for evaluating responsible vessels, the severity of stenosis and treatment outcomes in ischemic cerebral infarction (ICI) [1, 2].

Achieving homogeneous and high vascular contrast enhancement is essential for accurate interpretation of abnormal vascular findings [3]. The application of low-keV techniques in dual-energy CT effectively enhances vascular contrast by reconstructing low keV virtual monoenergetic images (VMI), essentially creating a CT image based on the attenuation of low-energy X-rays, which aligns more closely with the K-edge of iodine (33.2 keV), resulting in stronger attenuation and higher contrast [4]. This method typically requires lower contrast dosage or flow rate, making it more patient-friendly for those with chronic kidney disease [5]. However, the low energy X-rays in these images can lead to significant attenuation and reduced signal, contributing to high image noise that may fall short of clinical standard [6].

With the advancement of artificial intelligence technology, there has been an increasing use of deep learning techniques for reconstructing CT images [7, 8]. Deep learning image reconstruction algorithms (DLIR) have been shown to reduce noise and enhance diagnostic in low-dose conditions. This algorithm is increasingly applied in diverse clinical contexts, including ultra-low-dose lung cancer screening, “double-low” CTA, and abdominal imaging [9–11]. Recently developed DLIR algorithm capable of reconstructing Gemstone Spectral Imaging (GSI) CT images has demonstrated low noise level and high image quality without compromising texture, as shown in phantom and clinical studies [12–13]. Thus, in this study, the feasibility in the improvement of image quality and diagnostic confidence in head and neck CTA was systematically investigated by employing DLIR-GSI algorithm in dual-energy CT (DECT), especially with low contrast dose.

## Materials and methods

### Participants

**Fig. 1:**
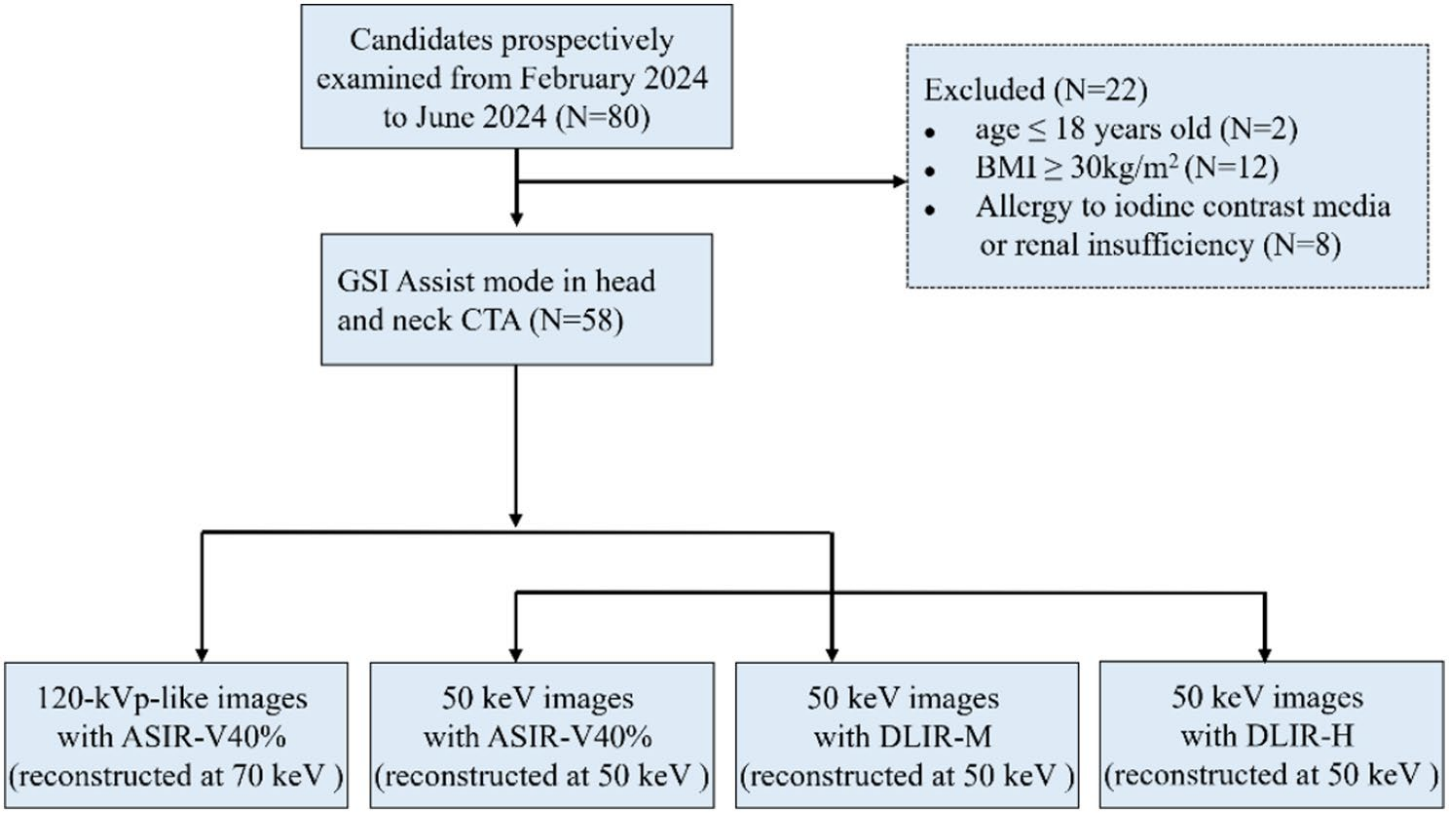
Flowchart of the study design. GSI, Gemstone Spectral Imaging; BMI, body mass index; ASIR-V, adaptive statistical iterative reconstruction-Veo; DLIR-M, deep learning image reconstruction at medium strength; DLIR-H, deep learning image reconstruction at high strength

This prospective study was approved by the Institutional Review Board in our hospital (XYFY2024-KL456-01), and all patients provided informed consent. Adult patients underwent head and neck CTA examination in our hospital from February 2024 to June 2024 were recruited in this study. Patients who met any of the following criteria were excluded: (I) age ≤ 18 years old (II) body mass index (BMI) ≥ 30 kg/m^2^. (III) allergy to contrast agents (IV) potential pregnancy (V) impaired renal function, hyperthyroidism, psychiatric disorder (Fig. 1).

### Scanning protocols

All participants were scanned on a 256-row Revolution Apex CT scanner (GE Healthcare, USA). The scans were performed with GSI Assist mode using rapid kV switching of 80/140 kVp. The acquisition parameters are present in Table 1. Patients were in the natural supine position with calm breathing and the range of scan was from the aortic arch (AA) to the top of the skull. The bolus-tracking technique was used with the monitoring region of interest (ROI) set in the ascending aorta under a trigger threshold of 100 HU. Nonionic contrast media (350 mgI/mL, Omnipaque 350, GE Healthcare, Shanghai, China) was injected with a high-pressure syringe (CT Motion, Ulrich Medical, Germany) through the right median cubital vein. Contrast media volume was adapted to the body mass index (BMI): A total of 30 mL contrast media was injected at a rate of 4.0 mL/s followed by a 30-mL saline chaser at a same rate when patient’s BMI was between 25 kg/m^2^ and 30 kg/m^2^. A bolus of 25 mL contrast media was injected at a rate of 3.5 mL/s and followed by 30 mL saline flush at the same injection rate when the patient’s BMI was less than 25 kg/m^2^.

**Table 1:** Scanning parameters

Parameters	Value
Tube voltage (kV)	80/140
Tube current (mA)	GSI-Assist (300–400 mA)
Noise index (HU)	10 *
Helical Pitch	0.984:1
Rotating time (seconds)	0.5
Detector collimation (mm)	128 × 0.625
Slice thickness (mm)	5
Kernel	Standard (ASIR-V40%) None (DLIR) ^#^

Note: *, corresponds to ASIR-V40%. ^#^, The DLIR algorithm does not have a reconstruction kernel

### Imaging reconstruction

70 keV VMIs were reconstructed with 0.625 mm slice thickness to create 120-kVp-like images for DECT scans, since the CT attenuation of images at 70 keV were reported to be comparable to those of 120-kVp images [14, 15]. And other VMI were reconstructed at 50 keV with 0.625 mm slice thickness employed three reconstruction algorithms: ASIR-V at 40% blending levels, DLIR at medium (DLIR-M), and high (DLIR-H) strength. Four groups of images (120-kVp-like with ASIR-V40%, 50 keV with ASIR-V40%, 50 keV with DLIR-M, 50 keV with DLIR-H) were compared with quantitative and qualitative analysis.

### Quantitative analysis

All reconstructed images were processed into the GSI Viewer software (Advantage Workstation, version 4.7, GE Healthcare) for further analysis. All measurements were collected by a radiologist with 15 years of experience in carotid radiology. The image noise, signal-to-noise ratio (SNR), contrast-to-noise ratio (CNR) and the margin sharpness were assessed on different reconstruction algorithms images.

### Image noise

For each group, the standard deviation (SD) of the attenuation value in a circular ROI with a diameter of approximately 5 mm placed in the sternocleidomastoid muscle (SCM) at the level of epiglottis and the cerebral white matter (WM) at the level of splenium of corpus callosum were recorded for evaluating the neck and head image noise respectively. In addition, the CT attenuations and SDs of the aortic arch (AA), bifurcation of the common carotid artery (CCA), origin of the internal carotid artery (ICA), M1 segment of the middle cerebral artery (MCA) were measured. ROIs were placed in the central parts of the arteries as much as possible while avoiding arterial wall, plaques, and severe artifacts. The clone functions were used for ROI placement so that the same areas of ROIs could be drawn in the same location on each reconstruction.

### SNR and CNR

SNR and CNR of AA, CCA, ICA, M1 segment of MCA in each group were calculated. The SNR was obtained by dividing the CT attenuation of the target vessels by the image noise, as follows: SNR = HU _vessel_ / SD _vessel_. Furthermore, the CNR of the head (CNR _head_) and neck (CNR _neck_) were obtained, respectively, as follows: CNR _head_ = (HU _vessel_ –HU _WM_) / SD _WM_. CNR _neck_ = (HU _vessel_ − HU _SCM_) / SD _SCM_.

### Margin sharpness of the arteries

**Fig. 2:**
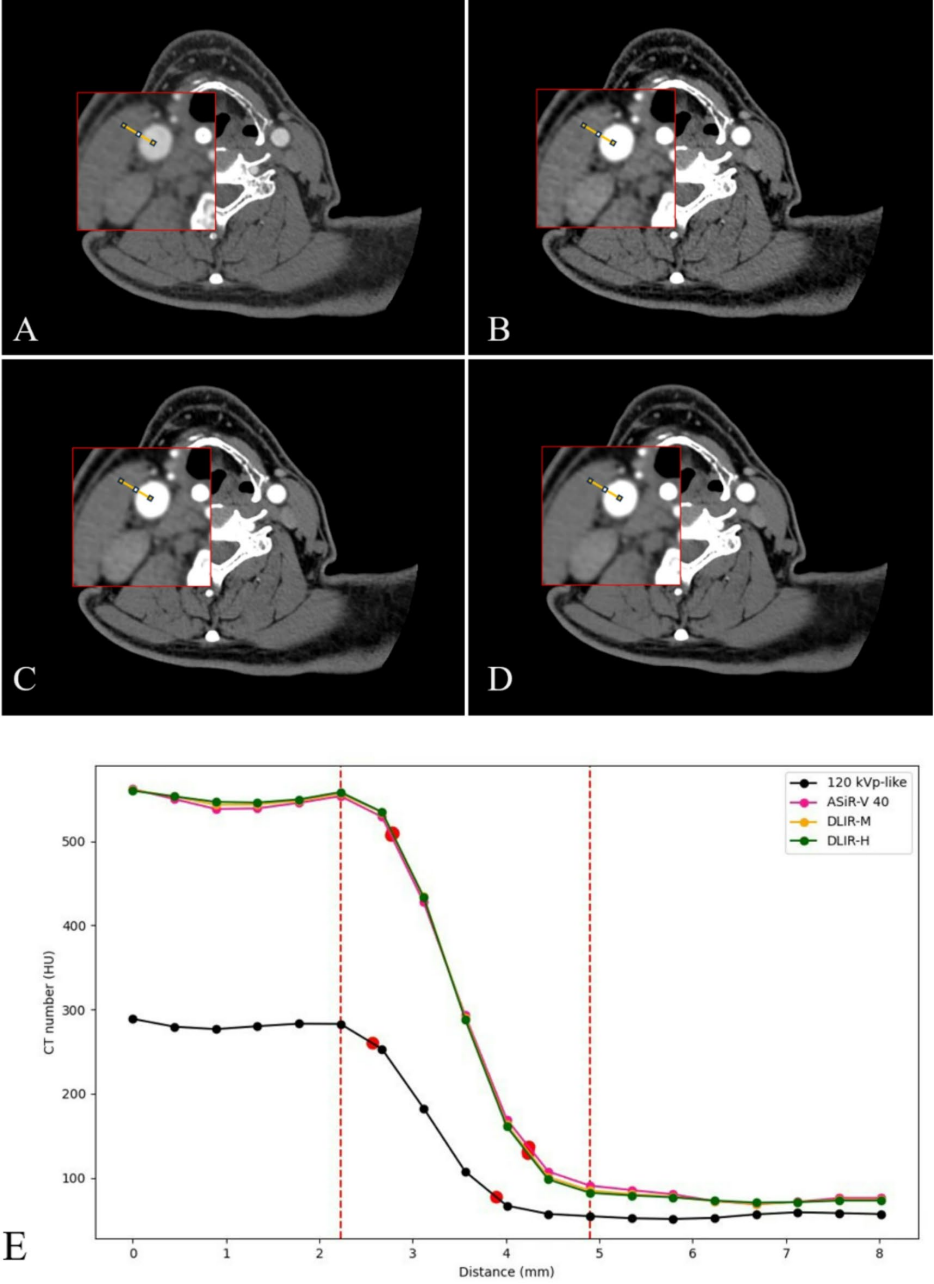
Examples of quantitative measurement used in our study. (**A**) reconstructed images from 120-kVp-like with ASIR-V40%; (**B**) reconstructed images from 50 keV with ASIR-V40%; (**C**) reconstructed images from 50 keV with DLIR-M; (**D**) reconstructed images from 50 keV with DLIR-H; E, ERD and ERS of four reconstruction algorithms. The common carotid artery running in the axial plane was selected, and a perpendicular line was drawn (the yellow lines in A - D), edge line profile was calculated reflecting the CT attenuation profile of those lines. In figure E, the red dashed lines in figure E indicated the last dip point and the first peak point on the rapid descending CT attenuation curve, and the red dots indicated the 10% point and 90% point. ERD was the distance between two red dots in its CT attenuation curve and ERS was the slope between these two red dots

CT attenuation profiles along a horizontal line through the lumen center of the right common carotid artery (Fig. 2A-D) were generated using the ImageJ software (National Institutes of Health, Bethesda, MD) and its particle analysis tool (Plot Profile), avoiding calcifications and plaques carefully. The clone functions were employed in the CT attenuation profiles generating process to keep the positions of the start and end points of the profiles in each reconstruction image consistent. Afterwards, the width of the edge response at the boundary of the common carotid arteries determined by the 10–90% edge rise distance (ERD) was measured, and the edge rise slope (ERS) was also calculated as follows: ERS = (HU 90% - HU 10%) / ERD. See Fig. 2(E).

### Qualitative analysis

The subjective quality of images was assessed by two experienced radiologists (with 10 and 15 years of experience in CTA diagnosis) blindly and independently and graded the image quality with a window width of 400 HU and a window level of 40 HU in terms of overall image quality, edge sharpness of vessels, image noise, artifacts and diagnostic acceptability using a 5-point Likert scale (Table 2). Images with a subjective score greater than 3 were considered to meet the clinical diagnosis need. Notably, if a disagreement was observed between two readers, the average score of the two readers was used as the final image quality score.

**Table 2:** Grading scales for the qualitative image analysis

Score	Overall image quality	Sharpness	Noise	Artifacts
1	Non-Diagnostic and unacceptable	Blurry	Unacceptable	Severe
2	Poor	Poorer than average	Above average	Poor
3	Moderate	Average	Average	Moderate
4	Good	Better than average	Less-than average	Minor
5	Excellent	Sharpest	Minimal or no noise	Absent

### Statistical analysis

All data were analyzed using the SPSS statistical software (SPSS for Windows, version 21.0; SPSS). Continuous variables were presented as the mean ± SD, and the Kolmogorov-Smirnov was used to test their normality. The median with interquartile range or frequency was used to represent the nonparametric variables. The CT attenuation, image noise, SNR, CNR, ERD and ERS were normally distributed and compared using the one-way ANOVA with Bonferroni correction The image quality score of the subjective grading was compared using the Friedman test and the paired Wilcoxon signed-rank test. Spearman’s rank correlation test was used to assess the relationship between objective and subjective evaluation. A correlation coefficient between 0.8 and 1 indicates a very strong correlation, between 0.6 and 0.8 indicates a strong correlation, between 0.4 and 0.6 indicates a moderate correlation, between 0.2 and 0.4 indicates a weak correlation, and between 0.0 and 0.2 indicates a negligible or no correlation. The Cohen kappa coefficient was used to quantitively evaluate the inter-observer agreement, where the *κ* value of less than 0.20 = poor, 0.21–0.40 = fair, 0.41–0.60 = moderate, 0.61–0.80 = good, and 0.81-1.00 = excellent. *P* < 0.05 was considered to represent a statistically significant difference. The sample size was calculated by G Power 3.1 [16].

## Results

### Patients

58 patients were enrolled, 35 (60.3%) of whom were male and 23 (39.7%) were female, with an age of 61.8 ± 13.6 years, a BMI of 24.9 ± 2.4 kg/m^2^. The BMI-based contrast administration resulted in an iodine delivery rate (IDR) of 1.32 ± 0.88 gI/s with a total iodine load of 9.72 ± 0.88 gI.

### Objective image quality

**Fig. 3:**
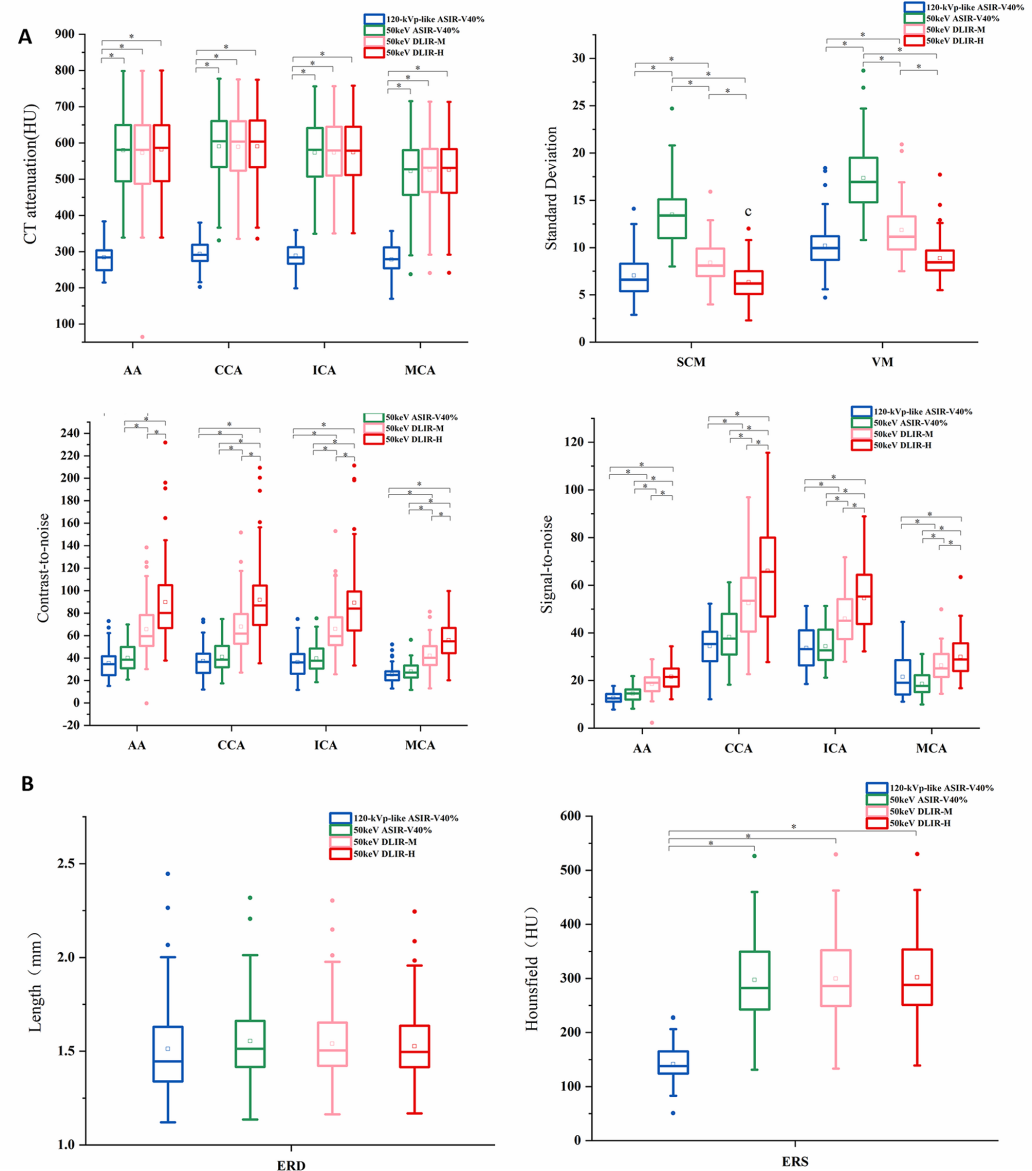
Comparison of the objective evaluation of the different reconstruction algorithms. (**A**) boxplot shows the CT attenuation, noise, SNR and CNR of the four groups (120-kVp-like with ASIR-V40%, 50 keV with ASIR-V40%, 50 keV with DLIR-M, 50 keV with DLIR-H). (**B**) comparison of the ERD and ERS among the four groups. * indicates *p* < 0.05

The CT attenuation of all vessels in the 120kVp-like images were significantly lower than the other three sets of 50 keV images (all *P* < 0.05), whereas the vascular CT attenuation in the 3 sets of 50 keV were comparable pair wise (all *P* > 0.05). In the 50 keV images, both SCM and WM had a minimum noise in DLIR-H group, and a maximum in ASIR-V40% group with significant difference (all *P* < 0.001). In addition, these two noises in the DLIR-H group were comparable to those in 120 kVp-like images (all *P* > 0.05). SNR and CNR in 50 keV images of all vessels had the same results: highest in DLIR-H group and lowest in ASIR-V40% group with significant differences (all *P* < 0.05). And the SNR and CNR in ASIR-V40% group were comparable to those in 120 kVp-like images (all *P* > 0.05). The mean value of ERD showed no significant difference among the four groups (*P =* 0.082). While the 120kVp-like images had the lowest ERS, which showed statistically significant difference with the other groups (all *P* < 0.001) (Table 3; Fig. 3).

**Table 3:** Objective image quality comparison among various reconstructions

Items	120kVp-like ASIR-V40%	50 keV ASIR-V40%	50 keV DLIR-M	50 keV DLIR-H	*p* value
**CT attenuation (HU)**					
AA	283.8 ± 39.14	579.9 ± 102.70	572.5 ± 122.67	581.8 ± 102.62	< 0.001
CCA	293.5 ± 35.68	590.2 ± 102.30	589.4 ± 101.86	590.3 ± 101.10	< 0.001
ICA	288.6 ± 37.31	572.9 ± 96.12	573.6 ± 96.10	574.0 ± 96.32	< 0.001
MCA	278.1 ± 40.95	522.5 ± 103.03	525.9 ± 102.88	525.7 ± 102.87	< 0.001
SCM	57.5 ± 7.14	69.6 ± 12.04	69.4 ± 12.30	70.2 ± 10.11	< 0.001
WM	36.8 ± 3.68	53.4 ± 8.01	53.2 ± 6.74	53.4 ± 6.45	< 0.001
**Noise (HU)**					
AA	22.8 ± 3.41	40.9 ± 6.44	31.7 ± 5.76	27.8 ± 5.29	< 0.001
CCA	9.2 ± 2.92	16.4 ± 4.40	12.0 ± 3.44	9.7 ± 3.11	< 0.001
ICA	9.2 ± 2.84	17.3 ± 4.04	13.0 ± 3.32	11.0 ± 2.91	< 0.001
MCA	14.5 ± 4.87	29.0 ± 6.05	20.6 ± 4.27	18.2 ± 3.99	< 0.001
SCM	7.0 ± 2.41	13.5 ± 3.48	8.4 ± 2.51	6.3 ± 2.01	< 0.001
WM	10.2 ± 2.63	17.3 ± 3.67	11.9 ± 2.83	8.9 ± 2.15	< 0.001
**SNR**					
AA	12.7 ± 2.11	14.5 ± 3.20	18.6 ± 4.83	21.6 ± 5.11	< 0.001
CCA	34.5 ± 8.99	38.3 ± 10.94	52.4 ± 15.68	66.0 ± 20.78	< 0.001
ICA	33.6 ± 9.10	34.4 ± 7.80	45.9 ± 10.98	54.5 ± 12.95	< 0.001
MCA	21.5 ± 8.22	18.5 ± 4.55	26.3 ± 6.63	30.0 ± 8.49	< 0.001
**CNR**					
AA	35.5 ± 12.45	40.1 ± 13.01	65.6 ± 25.72	89.7 ± 37.98	< 0.001
CCA	37.3 ± 13.41	41.0 ± 13.26	67.9 ± 26.04	91.7 ± 38.55	< 0.001
ICA	36.5 ± 13.21	39.7 ± 13.02	65.8 ± 25.35	89.1 ± 38.43	< 0.001
MCA	25.2 ± 8.06	28.0 ± 7.87	42.1 ± 13.43	55.9 ± 17.28	< 0.001
**ERD (mm)**	1.51 ± 0.27	1.55 ± 0.23	1.54 ± 0.22	1.53 ± 0.21	0.802
**ERS(HU/mm)**	141.4 ± 35.07	297.2 ± 77.38	299.5 ± 77.18	301.8 ± 76.92	< 0.001

Data are presented as mean ± standard deviation. ASIR-V, adaptive statistical iterative reconstruction veo; DLIR-M, deep learning image reconstruction at medium weighting; DLIR-H, deep learning image reconstruction at high weighting; SNR, signal-to-noise ratio; CNR, contrast-to-noise ratio; AA, the aortic arch; CCA, the common carotid artery; ICA, the internal carotid artery; MCA, the middle cerebral artery; ERD, edge rise distance; ERS, edge rise slope

### Subjective image quality

**Fig. 4:**
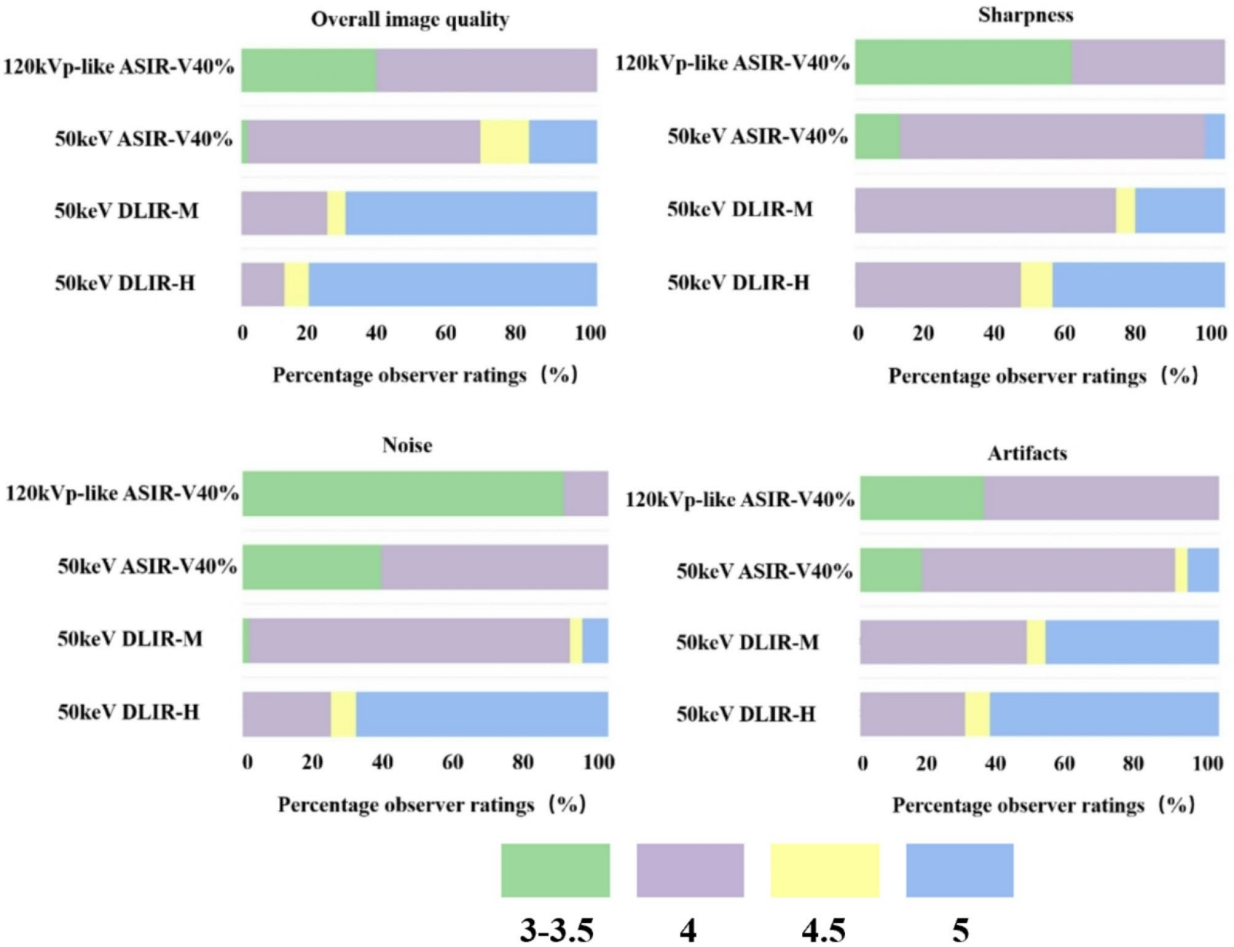
Stacked bar graph with ratings for subjective image quality criteria across all cases and observers

The summary of subjective image evaluation is shown in Table 4; Fig. 4. For overall image quality, sharpness, noise and artifacts, the 50 keV DLIR-H images received the highest scores, followed by the 50 keV DLIR-M and the 50 keV ASIR-V40, with the 120kVp-like ASIR-V40% images receiving the lowest scores. Great interobserver agreements were observed between the two readers with kappa values ranging from 0.84 to 0.92.

**Table 4:** Qualitative image quality assessment among various reconstructions

	120kVp-like ASIR-V40%	50 keV ASIR-V40%	50 keV DLIR-M	50 keV DLIR-H	*p1*	*p2*	*p3*	*p4*	*p5*	*p6*
Overall image quality	3.7±0.39	4.2±0.43	4.7±0.43	4.8±0.34	<0.001	<0.001	<0.001	<0.001	<0.001	1.000
Sharpness	3.5±0.48	3.9±0.38	4.3±0.43	4.5±0.48	<0.001	<0.001	<0.001	0.014	<0.001	0.142
Noise	3.1±0.34	3.7±0.46	4.1±0.28	4.7±0.43	<0.001	<0.001	<0.001	0.003	<0.001	<0.001
Artifacts	3.7±0.44	4.0±0.53	4.5±0.49	4.7±0.45	0.172	<0.001	<0.001	<0.001	<0.001	0.728
Average score	3.5±0.22	4.0±0.28	4.4±0.28	4.7±0.25	<0.001	<0.001	<0.001	<0.001	<0.001	0.011

*pl*,* p* value between 120kVp-likeASIR-V40% images and 50 keV ASIR-V40% images; *p*2, *p* value between 120kVp-likeASIR-V40% images and 50 keV DLIR-M images; *p3*,*p* value between 120kVp-like ASIR-V40% images and 50 keV DLIR-H images; *p4*, *p* value between 50 keV ASI.R-V40% images and 50 keV DLIR-M images; *p5*, *p* value between 50 keV ASIR-V40% images and 50 keV DLIR-H images; *p*6, *p* value between S0keV DLIR-M images and 50 keV DLIR-H images

### The correlation between objective and subjective indicators

ERS was moderately correlated with subjective sharpness scores, whereas the correlation between ERD and subjective sharpness scores did not reach statistical significance (*p* > 0.05). The SNR of the MCA and the CNR of the AA were moderately correlated with subjective noise scores, while the SNR and CNR of all other vessels were strongly correlated with subjective noise scores (Table 5).

**Table 5:** The correlation coefficient between objective and subjective indicators

Objective indicators	Correlation coefficient	*p* value
	Vs. subjective sharpness score	
ERD	0.043	0.513
ERS	0.421	< 0.001
	Vs. subjective noise score	
**SNR**		
AA	0.635	< 0.001
CCA	0.629	< 0.001
ICA	0.630	< 0.001
MCA	0.399	< 0.001
**CNR**		
AA	0.595	< 0.001
CCA	0.619	< 0.001
ICA	0.609	< 0.001
MCA	0.619	< 0.001

ERD, edge rise distance; ERS, edge rise slope; SNR, signal-to-noise ratio; CNR, contrast-to-noise ratio; AA, the aortic arch; CCA, the common carotid artery; ICA, the internal carotid artery; MCA, the middle cerebral artery

## Discussion

The present results demonstrated that the 50 keV monochromatic images reconstructed with DLIR-H exhibited lower image noise, as well as higher SNRs and CNRs, resulting in enhanced overall image quality compared to those reconstructed with DLIR-M or the 120-kVp-like images reconstructed with ASIR-V 40%.

In recent years, DECT has seen widespread use in clinical CTA examinations [17–19]. One of the commercial DECT platform is GSI, which employs rapid kVp-switching system to alternate tube voltages between 80 and 140 kVp. This system captures sinograms at two different energy and can reconstruct 101 VMIs range from 40 keV to 140 keV using DECT algorithm [6]. In low-energy VMIs, such as 40 or 50 keV, the lower X-ray energy is closer to the K-edge of the contrast agent (iodine), resulting in enhanced iodine attenuation and improved contrast [20]. Patino M et al. [21] reported that low-energy VMIs (40 and 50 keV) provided high diagnostic quality images for evaluation of the aortoiliac system, with intravascular attenuation increasing by 59–137%, while preserved CNR. In clinical practice, the target vascular attenuation for traditional CTA is typically 300–450 HU [22], ensuring sufficient contrast between vessels and surrounding tissues while avoiding interference with the display of calcifications. Additionally, in 50 keV CTA images, the CT attenuations of calcified plaques are also increased. In this study, the CT attenuations at 50 keV are slightly higher than the target range of traditional CT, which significantly increases the vascular contrast while not affecting the display of calcifications, and therefore meet the clinical diagnostic requirements. In our study, the CT attenuation for AA, CCA, ICA, and MCA on the 50 keV images increased by approximately 51% compared to those on the 120 kVp-like images. Our results also revealed that, compared to 120 kVp-like images, 50 keV monochromatic images significantly enhanced vascular visibility and provided significantly higher CT attenuation.

However, low-keV X-rays undergo greater attenuation in biological tissues, resulting in decreased signal and increased noise levels in the images [23]. Traditional iterative reconstruction (IR) algorithms, such as ASIR-V, greatly reduced the noise in low keV images, but higher IR intensities still result in suboptimal image texture, spatial resolution, and lesion detection capabilities [24]. DLIR technique overcomes the limitations of both conventional and iterative reconstruction. By using deep convolution neural networks (DCNN), DLIR models are trained to process low-dose CT projections and produce reconstructed images that closely resemble high-dose images generated by the Filtered Back Projection (FBP) algorithm [25]. Due to its distinct advantages, DLIR has been incorporated into GSI applications to further reduce noise and improve image texture. DLIR for GSI builds upon the extremely fast switching speed of the generator and fast response of the Gemstone detector to conserve spatial sampling with twice the number of samples relative to single-energy combined with DLIR reconstruction to manage image noise, texture and artifacts [26]. According to previous studies, for both natural image texture and noise reduction magnitude, the image quality has been proven generally equivalent between DLIR for single-energy and GSI dual-energy [27].

There are many studies [12, 28, 29] on the combination of conventional CT and deep learning-based reconstruction algorithms, and there are also some in the field of head and neck CTA [24, 30], mainly focus on conventional CT images (120kVp, 100kVp, 80kVp, 70kVp images, etc.). Our work explores the application of DECT combined with DLIR algorithms in head and neck CTA to improve the imaging results at low keV images (50 keV). Previous studies have already confirmed the clinical value of DLIR in GSI applications. Fukutomi et al. [31]. reported that DLIR combined with GSI could substantially reduce image noise in an abdominal clinical study compared to other reconstruction algorithms. Jiang et al. [32]. showed DLIR-H significantly improve image quality in clinical carotid DECTA compared to ASIR-V 80%, while maintaining a desirable diagnostic performance and arterial depiction. DLIR with three selectable strength levels (Low, Medium, High) can be built into the reconstruction protocols based on the clinical applications and radiologist preference. Notably, several studies [33, 34] illustrated a reduction in image noise and an increase in CNR as a function of DLIR strength. In our study adopted a 40% ASIR-V blending factor as reference standard, due to the more common use of ASIR-V 40% in clinical practice. DLIR provided superior noise reduction compared to ASIR-V 40%, with image noise decreasing progressively as the DLIR strength increased. At 50 keV, the image noise level for DLIR-H was comparable to that of 120 kVp-like ASIR-V 40%, indicating that DLIR-H effectively compensates for the noise increase associated with lower X-ray energy. Furthermore, while the 50 keV images reconstructed with ASIR-V 40% did not show significant improvements in SNR and CNR compared to 120 kVp-like images, the use of DLIR led to notable enhancements in both metrics, with the highest improvements observed at the strongest DLIR strength (DLIR-H). Additionally, correlation analysis showed that readers’ perception of image noise was strongly correlated with the objective image quality represented by CNR and SNR.

In our study, we extended the objective measurements to include the evaluation of edge sharpness across different reconstructions. ERD and ERS of the common carotid arteries were used to further objectively assess the effect of reconstruction algorithms on image spatial resolution and edge sharpness. Our results showed that there was no significant difference in the ERD among the four groups. The ERS value of image groups at the 50 keV was similar, but were all higher than that of the 120kVp-like images with a significant difference (all *P* < 0.05). The reconstruction algorithm and X-ray energy do not influence the distance required for CT attenuation to increase from 10 to 90% at the object edges (ERD). However, when using slope to represent sharpness, both distance and CT attenuation play a role. In comparison to 120 kVp-like images, the CT attenuation at the object edges in 50 keV images show a significantly greater increase, leading to much higher ERS values. We contend that the slope (ERS) is a better indicator of sharpness, suggesting that sharpness is improved in the three groups at 50 keV. This also indicates that using low keV images from DECT can enhance the edge sharpness of head and neck vessels. Furthermore, the DLIR-H group received the highest subjective scores in terms of the image noise and average score, which was consistent with previous studies that the image noise was significantly reduced, and the image quality and diagnostic performance were improved.

Moreover, different from previous studies on the head and neck CTA, we implemented a customized contrast injection protocol based on the patient’s BMI. Our results revealed that the mean IDR was 1.32 gI/s and the mean total iodine load was 9.72 gI, both lower than those recommended in the guidelines [35]. Combined with the use of the DLIR algorithms in DECT, this customized contrast injection protocol not only provided superior image quality compared to conventional scanning but also reduced medication risk for patients, further promoting the applicability of head and neck CTA. The present findings showed that the DLIR algorithm could produce superior image quality with contrast medium doses, as well as reduced injection rate thereby enhancing patient comfort.

There are still some limitations reserved in this study. First, our study population was relatively small, and further investigations involving larger cohorts are ongoing to validate our preliminary findings. Secondly, our study only compared 40%ASIR-V and DLIR algorithms, without including higher levels of ASIR-V for comparison. Finally, patients with BMI out of the normal range were excluded from this study. Future studies will be carried out to examine the effectiveness and applicability of this CTA protocol across a broader patient population.

## Conclusion

In this study, DLIR has been proven a promising applicable technology for DECTA reconstruction, since it can significantly reduce image noise, promoting the improvement of head and neck DECTA at 50 keV. It is worth considering adopting in routine head and neck CTA applications.

## Electronic supplementary material

Below is the link to the electronic supplementary material.

Supplementary Material 1

## Data Availability

The datasets used and/or analysed during the current study are available from the corresponding author on reasonable request.

## Abbreviations

CTA: Computed tomography angiography
ICI: Ischemic cerebral infarction
DLIR: Deep learning imaging reconstruction
GSI: Gemstone Spectral Imaging
DECTA: Dual-energy computed tomography angiography
ASIR-V: Adaptive statistical iterative reconstruction-Veo
DLIR-H: DLIR with high settings
DLIR-M: DLIR with medium settings
SNR: Signal-to-noise ratio
CNR: Contrast-to-noise ratio
DECT: Dual-energy CT
BMI: Body mass index
AA: Aortic arch
ROI: Region of interest
VMI: Virtual monochromatic images
SCM: Sternocleidomastoid muscle
WM: White matter
CCA: Common carotid artery
ICA: Internal carotid artery
MCA: M1 segment of the middle cerebral artery
ERD: Edge rise distance
ERS: Edge-rise slope
IDR: Iodine delivery rate
IR: Iterative reconstruction
DCNN: Deep convolution neural networks
FBP: Filtered back projection
