# Supplementary material for: Image quality improvement in head and neck angiography based on dual-energy CT and deep learning
Source: BMC Med Imaging. 2025 Apr 10;25:115. doi: 10.1186/s12880-025-01659-4 (PMC11987473; doi:10.1186/s12880-025-01659-4)
Supplement: Supplementary file 1 — Supplementary Material 1 [file 12880_2025_1659_MOESM1_ESM.pdf]

Supplementary Files

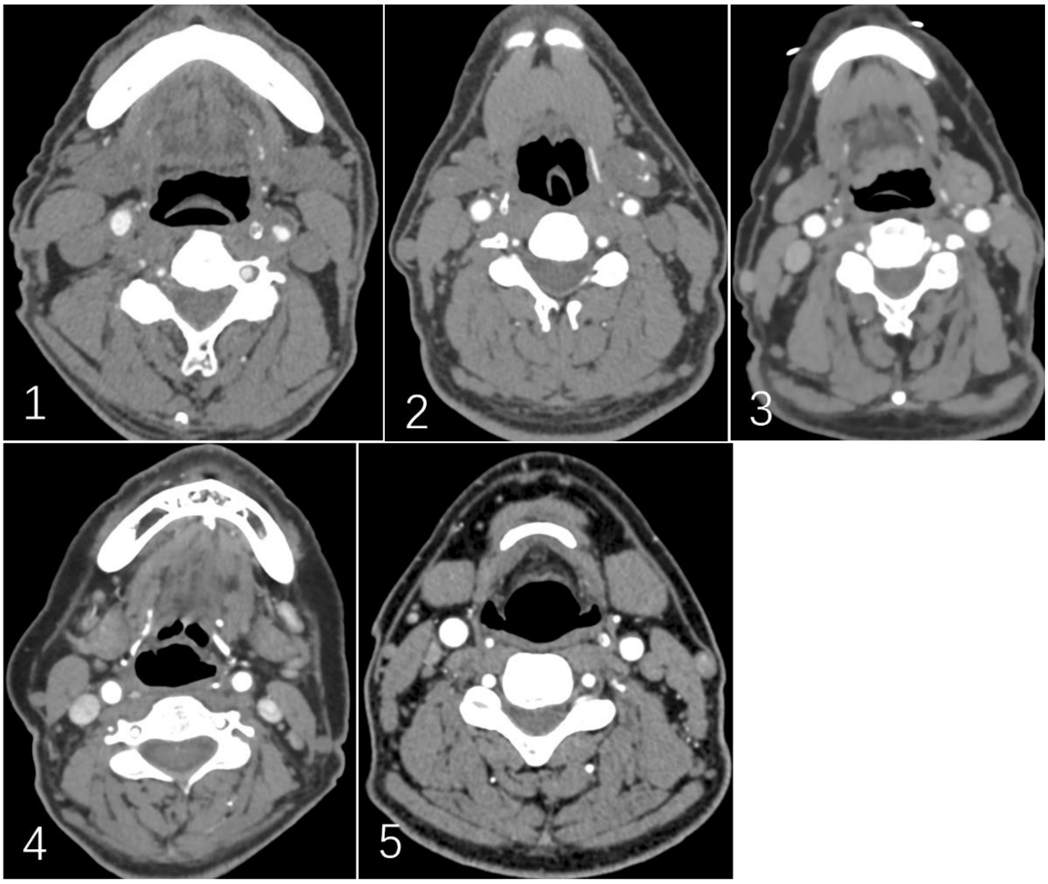

Figure 1 Example images of sharpness scores ranging from 1 to 5. **1**, Score of 1. **2**, Score of 2. **3**, Score of 3. **4**, Score of 4. **5**, Score of 5.

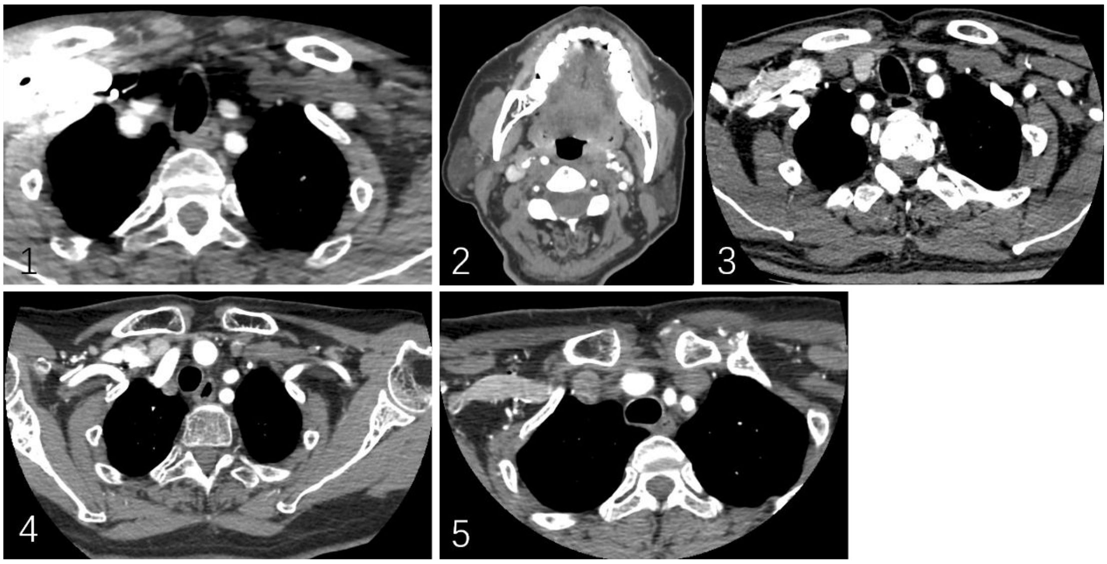

Figure 2 Example images of artifacts scores ranging from 1 to 5. **1**, Score of 1. **2**, Score of 2. **3**, Score of 3. **4**, Score of 4. **5**, Score of 5.

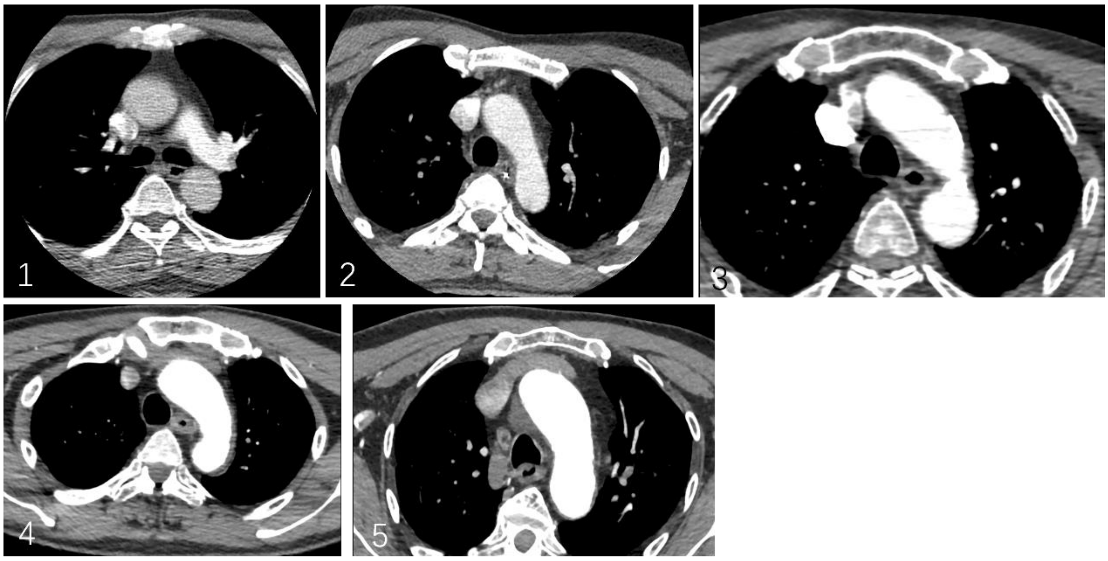

Figure 3 Example images of noise scores ranging from 1 to 5. **1**, Score of 1. **2**, Score of 2. **3**, Score of 3. **4**, Score of 4. **5**, Score of 5.

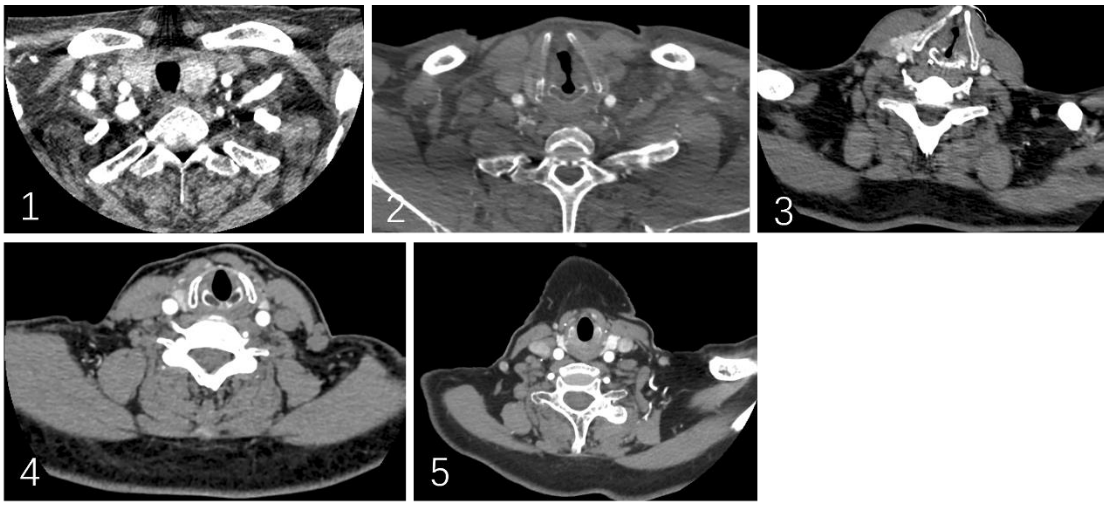

Figure 4 Example images of overall image quality scores ranging from 1 to 5. **1**, Score of 1. **2**, Score of 2. **3**, Score of 3. **4**, Score of 4. **5**, Score of 5.
